# INTEREST GROUP SESSION—AGING AMONG ASIANS: RESEARCH METHODS IN AGING AMONG ASIANS

**DOI:** 10.1093/geroni/igz038.1329

**Published:** 2019-11-08

**Authors:** Wenjun Li, Shantha Balaswamy, Allen Glicksman

**Affiliations:** 1 University of Massachusetts Medical School, Worcester, Massachusetts, United States; 2 The Ohio State University, Columbus, Ohio, United States; 3 Philadelphia Corporation for Aging, Philadelphia, Pennsylvania, United States

## Abstract

Asians are the largest and the fastest growing segment of the world population. Asian immigrants are the second largest immigrant population in the U.S. However, age-related social and health issues are understudied among late-life immigrant and the oldest old Asians. Little data exist to support public health promotion, policy studies and clinical practice in this population. To advance research into aging among Asians living in the U.S. and elsewhere in the world, sound methodologies can be adopted from those well-developed in other settings while novel methodologies are to be developed to meet the unique needs of Asian studies. This symposium brings together four abstracts that address a variety of common methodological issues in social and health studies among Asian older adults. The topics range from culturally and linguistically appropriate strategies for recruiting non-English speaking research participants, assessment of social isolation and transportation barriers using an ethnographical approach, development of a new culturally appropriate measure for successful aging among the oldest old Chinese in China, and evaluation of preventive healthcare use among faith-based first-generation Chinese immigrants using self-administered surveys in the U.S. These studies involve qualitative ethnographical analysis, mixed methods for instrument development, quantitative data analysis, use of geographic information systems and demography to plan participant recruitment, and use of staged community engagement to increase efficiency and representativeness of participant recruitment. Lessons learned from these studies are valuable to future studies on aging among Asians. This symposium is a collaborative effort of the GSA Aging Among Asians Interest Group.

